# Longitudinal trajectories of eating disorder symptoms in mothers: the predictive role of PTSD and childhood trauma

**DOI:** 10.3389/fpsyg.2025.1565102

**Published:** 2025-10-01

**Authors:** Sofie Egidius Helle, Pernille Telstad Wangsmo, Line Indrevoll Stänicke, KariAnne R. Vrabel

**Affiliations:** ^1^Department of Psychology, University of Oslo, Oslo, Norway; ^2^Lovisenberg Hospital, Nic Waals Institute, Oslo, Norway; ^3^Modum Bad, Research Institute, Vikersund, Norway

**Keywords:** eating disorders, motherhood, post-traumatic stress disorders, psychotherapy, trauma

## Abstract

**Objective:**

A significant sub-group of patients with eating disorders (ED) are mothers. There is limited literature on this population, and little is known about their treatment trajectories and outcomes. The primary objectives of this study were to examine; (1) longitudinal changes in ED symptomatology among mothers who underwent a specialized sequential treatment, and (2) the extent to which post-traumatic stress disorder (PTSD) and/or childhood trauma predicted treatment outcome.

**Methods:**

A total of 61 patients with ED received a highly specialized, sequential inpatient Cognitive Behavior Therapy (CBT) program for mothers delivered over 6 weeks in total, with one treatment week per month across a six-month period. ED symptoms were measured with Eating Disorder Examination-questionnaire (EDEQ) at admission, discharge, and 1-year follow-up. Data were analyzed with multi-level analysis, and we examined outcomes for both overall symptom reduction, as well as for the four clinical subscales that EDE is comprised of, e.g., “Weight concern,” Shape concern, Eating Concern, and Restriction.

**Results:**

There was a significant reduction in ED symptoms from admission to discharge, which remained stable through the 1-year follow-up. PTSD and childhood trauma predicted the level of “Shape concern” negatively across the entire period. PTSD also predicted the level of “Weight concern” negatively over time.

**Discussion:**

Specialized sequential treatment for mothers with ED shows potential role as a promising intervention. However, further research is needed to confirm its effectiveness. Additionally, this study enhances our understanding of the role of trauma in the treatment of ED.

## Introduction

Eating disorders (ED) are expressed through serious and persistent disturbances in eating behavior, such as over- or under-controlled eating, extreme controlling behavior related to weight and body, and disturbances of bodily perceptions (Fairburn, 2008). The condition is associated with a high degree of psychiatric comorbidity (Bakalar et al., 2015; Keski-Rahkonen and Mustelin, 2016) and harmful neurological and somatic sequelae are common (Frostad, 2004; Gibson et al., 2019). In a socio-economic perspective, ED is associated with a significantly higher yearly public health cost and higher rates of unemployment compared to the general population (Stokke et al., 2022; Streatfeild et al., 2021). Cognitive behavioral therapy (CBT) (Waller et al., 2007) is considered the treatment of choice for reducing ED symptoms (National Institute for Health and Care Excellence, 2020) but up to half of patients do not recover after evidence-based treatment (Steinhausen, 2009; Treasure et al., 2020).

Most individuals diagnosed with ED have traditionally been women (Hoek, 2017), and despite concerns about fertility and pregnancy, a substantial proportion of individuals receiving treatment for an ED are mothers or mothers-to-be (Bryant-Waugh et al., 2007). Ongoing ED may impact parental functioning in various ways, including heightened preoccupation with body image, feelings of guilt, fear of intergenerational transmission of disordered eating behaviors, and difficulties with emotional attunement or availability (Allen et al., 2014; Claydon et al., 2018). Mothers with ED encounter multiple barriers to accessing care, including fear of stigma and disclosure (Claydon et al., 2018), concerns about custody or judgment from professionals, and practical difficulties balancing treatment demands with parenting responsibilities (Chapman et al., 2021). There is a lack of research on the specific needs and challenges faced by mothers with ED, as well as what they perceive as relevant and helpful support (Kaspersen and Rø, 2012; Sadeh-Sharvit and Lock, 2018; Sommerfeldt et al., 2022). To our knowledge, no treatment studies have yet been published that specifically examine this population. Consequently, we know too little about the symptom trajectories for ED symptoms or potential predictors for outcomes. Evidence from prior studies suggests that specialized, evidence-based interventions, such as cognitive behavioral therapy (CBT), are effective in reducing ED symptoms over time (Fairburn et al., 2015; Waller et al., 2007), both for relative and absolute outcomes (Cuijpers et al., 2024). In particular enhanced versions of CBT, such as inpatient programs, show promise as an effective form of intervention (Atwood and Friedman, 2020; de Boer et al., 2023). A recent critical synthesis of 44 systematic reviews on the effect of cognitive behavioral theory for ED confirmed the benefits for ED-related outcomes, especially when delivered with high intensity (Kaidesoja et al., 2023). Effective treatment for this group is particularly critical given that children of mothers with ED are at increased risk for negative developmental outcomes in various domains (Martini et al., 2020; Watson et al., 2018), including a heightened risk of developing eating problems themselves (Bould et al., 2015).

Although there is limited knowledge regarding the efficacy of treatment for women with ED in a parenting role, studies on ED treatment in general suggest that a subset of patients may be at higher risk for poor treatment outcomes (Vall and Wade, 2015). The combination of factors predicting symptom trajectories is complex, and remains poorly understood for ED in general (Linardon et al., 2017), with even less known about mothers with ED. However, existing evidence indicates that trauma experiences are often linked to ED pathology, both increasing the risk of developing an ED (Brewerton, 2007), and being associated with more serious ED symptoms compared to those without such experiences (Backholm et al., 2013; Molendijk et al., 2017). Untreated trauma, particularly from childhood, is associated with long-term impairments in parenting capacity, including increased stress, emotional dysregulation, and difficulties in forming secure attachments with their children (Madigan et al., 2006). Mothers with both ED and unresolved trauma may therefore face a compounded burden that impacts both their own recovery and their ability to parent effectively. Research has consistently shown that trauma, particularly childhood maltreatment, can exacerbate the chronicity and severity of ED symptoms (Brewerton, 2007; Molendijk et al., 2017). Traumatic experiences are thought to interfere with emotional regulation and coping mechanisms, which are critical for recovery from ED (Monteleone et al., 2018). In particular, PTSD has been linked to increased emotional dysregulation, which may perpetuate maladaptive eating behaviors as a way of coping with distress (Mitchell et al., 2021). Therefore, trauma-related factors such as PTSD and childhood trauma are likely to influence the long-term trajectory of ED symptoms, making it critical to assess their predictive value for treatment outcomes (Backholm et al., 2013).

Research has demonstrated that comorbid PTSD is associated with increased psychopathology in patients with ED. For instance, studies have found that individuals reporting more PTSD symptoms also experience more serious and pervasive ED symptoms (Day et al., 2024; Tagay et al., 2014). In a large longitudinal population study, Mitchell et al. (2016) found that a PTSD diagnosis predicted a higher degree of ED at a three-year follow-up. More recent findings suggest that PTSD is linked to poorer treatment outcomes (Hazzard et al., 2021), a greater likelihood of early treatment termination (Trottier, 2020) and higher relapse rates (Brewerton et al., 2023).

Furthermore, experiences of physical, emotional, or sexual abuse during childhood, as well as emotional and physical neglect, have been strong associated with the development of ED (Caslini et al., 2016; Kong and Bernstein, 2009; Molendijk et al., 2017; Palmisano et al., 2016; Pignatelli et al., 2017; Vidaña et al., 2020). Research suggests that repeated traumatic experiences early in life can result in more complex consequences than those captured by a PTSD diagnosis (Brewerton, 2022) with a clear association between the number of traumatic experiences reported and the severity of ED pathology (Groth et al., 2019). In particular, weight-related abuse in childhood, such as teasing, bullying, or shaming focused on body size or eating behaviors, has been identified as a distinct form of trauma that may contribute to the maintenance of ED symptoms over time. These experiences are associated with increased body shame, negative body image, and emotion regulation difficulties, which may in turn reinforce disordered eating as a coping mechanism (O’Loghlen et al., 2023; Puhl et al., 2007). For example, O’Loghlen et al. (2023) found that emotional maltreatment targeting body image in childhood predicted binge eating through increased body shame. Similarly, data from large population-based studies show that weight-related teasing during youth is associated with disordered eating in adolescence and adulthood (Puhl and Luedicke, 2012). Including such forms of interpersonal trauma in the assessment of childhood adversity may be particularly important when working with mothers, given the heightened societal scrutiny of maternal bodies and eating behaviors.

Despite extensive literature indicating a strong relationship between trauma and ED (Brewerton, 2007; Molendijk et al., 2017) these variables are understudied as predictors of treatment trajectories in ED, and identifying robust replicable predictors remains challenging (Linardon et al., 2017). Evidence shows that both the presence of PTSD and childhood traumatic experiences, are associated with poorer treatment outcomes for ED (Convertino and Mendoza, 2023; Day et al., 2023; Molendijk et al., 2017; Scharff, 2021). More longitudinal studies are needed to clarify illness trajectories and associated variables which will ultimately inform and improve treatment services (Marks, 2019). Given the general association between traumatic experiences and ED it is important to investigate these forms for traumatic experience, regardless of whether the individual meet the criteria for a diagnosis of PTSD.

In the light of limited research on mothers with ED undergoing therapy, there is a pressing need to examine the trajectories of ED symptoms over time, and to investigate relevant clinical predictors for this group. Given the established association between traumatic experiences and ED, it is particularly important to explore trauma as a predictor, regardless of whether the individual meets the diagnostic criteria for PTSD. Therefore, the main objectives of this study were therefore to explore; (1) longitudinal changes in ED symptomatology among mothers who had undergone specialized sequential inpatient treatment, and (2) whether PTSD and/or childhood trauma would predict ED trajectories over time. Specifically, we hypothesize the following:

Hypothesis 1: There will be a significant reduction in ED symptoms from admission to discharge, and these changes remaining stable at 1-year follow-up.

Hypothesis 2: A diagnosis of PTSD or childhood trauma at admission will negatively predict the trajectory of ED symptoms over time.

## Method

### Design

Participants were referred to treatment at the Department of Eating Disorders at Modum Bad psychiatric hospital in Norway from a local general practitioner’s office or a local psychiatric hospital. The clinic is a specialized hospital running an inpatient treatment program for patients with longstanding ED and a history of failing to respond to treatment in local treatment facilities. The unit treats anorexia nervosa (AN), bulimia nervosa (BN), and other feeding and eating disorders (OSFED). All patients in this study participated in a pre-care evaluation before admission and responded to questionnaires (see “Instruments”) related to ED symptoms at admission, discharge and 1-year follow up. Data were collected between 2011 and 2013. The specialized sequential treatment used during this period is still representative of many treatment models in use today. Therefore, the data provide insights into the effectiveness of a type of intervention that continues to be relevant, and the findings can still inform current treatment practices. Questionnaires were administered on paper in private during the inpatient weeks, and patients completed them independently. Staff were available to assist if needed. Completed forms were securely stored and later anonymized for research use.

### Treatment

The treatment consisted of an inpatient sequential, specialized program for mothers with ED (Kaspersen and Rø, 2012). The primary goals of the treatment were to normalize eating patterns, achieve a healthy weight, reduce compensatory behaviors, enhance emotion regulation skills, and strengthen awareness and competence in the parental role. The treatment team comprised five CBT-oriented clinicians: a psychologist, a psychiatrist, a social worker, and two psychiatric nurses. Treatment was delivered in intervals, with 1 week of inpatient treatment per month, over a six-month period. Although we do not have access to detailed information about the frequency or content of this follow-up care, all patients were required to remain in contact with their local outpatient clinic and general practitioner throughout the treatment period. This ensured continuity of care and allowed for coordination between inpatient and local services, although the exact nature of the support may have varied depending on local resources and patient needs. The therapeutic approach was based on a cognitive model of treatment (Waller et al., 2007) using a transdiagnostic framework (Fairburn et al., 2003). Each week of inpatient treatment included 4 h of individual CBT sessions. In addition to individual therapy patients participated in CBT-based group therapy, activity groups, and psychoeducational groups, totalling six group sessions per week. These sessions included two focused on physical activity and bodily mindfulness. Finally, a “Family Day” was incorporated where mother–child interactions were observed, and feedback was provided as part of the program. To ensure consistent delivery and treatment fidelity across all components, the intervention followed a structured and manualized format. All sessions were delivered by a dedicated multidisciplinary team with specialized expertise in ED treatment and consistent training in CBT principles. Weekly team meetings were held to discuss case progress and adherence to the treatment model. Furthermore, all treatment modules (individual CBT, group sessions, physical activity, and parental competence training) followed predefined schedules and content outlines. Although formal fidelity checklists were not used, the consistent staffing and structured approach supported a high level of treatment integrity across all treatment intervals.

### Participants

All patients who participated in the treatment program between 2011 and 2013 were included in this study (*N* = 61). All participants met the DSM-IV criteria for ED representing a transdiagnostic sample. The inclusion criteria for both the study and the treatment were a diagnosis of ED, one or more failed attempts at outpatient treatment and being a mother. Exclusion criteria included active psychosis, chronical suicidality, and ongoing substance abuse. Six patients (9.8%) were diagnosed with PTSD, while 20 (32.8%) had experienced at least one form for childhood trauma. The most common types of childhood trauma were sexual abuse (24.6%), physical abuse (13.1%), and emotional abuse (6.6%). A total of 12 patients experienced more than one type of trauma, on average, individuals reporting trauma had experienced approximately 1.53 types of trauma. Additionally, 19 patients (31.1%) reported current or past self-harm behavior. In addition to ED and PTSD diagnoses, the sample presented with various comorbidities, including affective disorders (64.0%), anxiety disorders (29.5%), personality disorders (1.6%), ADHD (3.3%), and somatoform disorders (4.9%). These findings reflect the clinical complexity and high burden of comorbid psychopathology in this population. Descriptive characteristics of the sample are provided in Table 1.

**Table 1:** Characteristics and demographic at admission.

Characteristics (*N* = 61)	
Sex	
Woman	61 (100)
Occupational status	
Employed, n (%)	22 (36.1)
Sick leave, n (%)	24 (39.3)
Disabled, n (%)	12 (19.7)
Maternity leave, n (%)	1 (1.6)
Student, n (%)	2 (3.3)
Civil status	
Single, n (%)	10 (6.1)
In a relationship, n (%)	1 (1.6)
Cohabitant, n (%)	17 (27.9)
Married, n (%)	26 (42.6)
Divorced, n (%)	7 (11.5)
Number of children	
One, n (%)	13 (21.3)
Two, n (%)	32 (52.5)
Three, n (%)	13 (21.3)
Four, n (%)	3 (4.9)
Age children, mean (SD)	8.6 (5.3)
Age mothers, mean (SD)	35.6 (6.9)
Age debut, mean (SD)	17.7 (5.1)
Duration of illness, mean (SD)	18.0 (8.2)
BMI, mean (SD)	22.1 (5.5)
Number of previous admissions, mean (SD)	1.3 (3.5)
Previous treatment, year, mean (SD)	4.00 (4.0)
Eating disorder diagnosis	
AN, n (%)	17 (27.9)
BN, n (%)	38 (62.3)
OSFED, n (%)	6 (9.8)
PTSD, n (%)	6 (9.8)
Childhood trauma, n (%)	20 (32.8)

n, numbers; SD, standard deviation; BMI, Body Mass Index; AN, Anorexia Nervosa; BN, Bulimia Nervosa; EDNOS, Eating Disorder Not Otherwise Specified; PTSD, Post-traumatic stress disorder.

### Patient flow

The original sample consisted of 61 patients of whom 52 completed treatment and 50 provided data at the 1-year follow up. For the nine participants who discontinued treatment prematurely, discharge measures were not consistently collected and are therefore not included in the discharge dataset. However, all available data from these participants at admission and follow-up were included in the analyses, consistent with the intention-to-treat approach. T-tests revealed no significant differences between completers and non-completers at baseline with respects to age (*M* = 36.0, *SD* = 7.0, and *M* = 33.3, *SD* = 6.0, respectively; *t*(59) = 1.1, *p* = 0.287), BMI (*M* = 22.4, *SD* = 5.8, and *M* = 20.1, *SD* = 2.9, respectively; *t*(59) = 1.2, *p* = 0.249), number of children (*M* = 2.1, *SD* = 0.8, and *M* = 2.2, *SD* = 0.7, respectively; *t*(59) = 0.6, *p* = 0.614), or *EDE global* mean score at time of admission (*M* = 3.9, *SD* = 1.2, and *M* = 3.8, *SD* = 0.8, respectively; *t*(32) = −0.0.2, *p* = 0.883).

### Instruments

#### Assessment

The diagnosis of an ED was based on three criteria. First, patients had to meet the diagnostic criteria for an ED according to the *Structural Clinical Interview for DSM-IV Axis I disorders* (First et al., 1997). This tool has demonstrated satisfactory psychometric abilities and is considered the gold standard for diagnosing Axis I conditions (Lobbestael et al., 2011). Second, all patients underwent an in-depth clinical interview. During this process, patients were weighted to obtain a measure of their weight and detailed questions were asked regarding the importance of body shape and weight for their self-esteem, their desire to lose weight and their specific eating rules. Third, the results on the Eating Disorder Examination-Questionnaire (EDE) were considered, with scores ≥2.50 regarded as above clinical cut-off (Rø et al., 2015).

#### Outcome

*Eating Disorder Examination-Questionnaire* (Bohn and Fairburn, 2008) was used to assess the severity of ED symptom. This self-report questionnaire is derived from the Eating Disorder Examination Interview (Cooper and Fairburn, 1987) and measures ED behaviors and symptoms. The EDE (Bohn and Fairburn, 2008) consists of four clinical subscales: “Restriction,” “Eating Concern,” “Weight Concern,” and “Shape Concern,” with each subscale represented by five to eight items. An EDE Global score is calculated based on these subscales The Norwegian version of the EDE translated by Rø et al. (2015) and has demonstrated good internal consistency and satisfactory test–retest reliability in studies conducted on adult populations (Rø et al., 2010). Additionally, it has shown discriminant validity between patient and controls (Rø et al., 2015). In this study, both the EDE Global score, as well as all subscales: “Restriction,” “Eating Concern,” “Weight Concern,” and “Eating Concern” were used as outcome variables.

#### Predictors

*SCID-I* (First et al., 1997) and a clinical interview were used to assess PTSD. Patients were coded 0 if they did not meet the criteria for PTSD, and 1 if they did.

*The Childhood Trauma Questionnaire – short form* (CTQ) was used to assess childhood trauma (Bernstein et al., 2003). CTQ measures childhood maltreatment in five areas, hence sexual, physical, and emotional abuse, as well as physical and emotional neglect. To identify potential cases of childhood trauma and differentiate between no trauma and trauma, we employed the categorical scoring system recommended by Walker et al. (1999). Patients were placed in the trauma group if they scored ≥8 on the sexual abuse, physical abuse, or physical neglect subscale; ≥10 on the emotional abuse subscale; or ≥15 on the emotional neglect subscale were placed in the trauma group. Based on the information from the anamnestic interview, three experienced therapists with clinical and research expertise in the field of ED coded the categorization of trauma cases. Patients were coded as 1 if they were considered to have experienced one or more types of maltreatment and as 0 if they did meet criteria for any of the five trauma categories.

### Statistical analysis

Statistical analyses were conducted using the Statistical Package for Social Sciences (SPSS, version 26.0). The analysis was based on repeated dimensional EDE scores collected at admission, discharge, and 1-year follow-up. Cohen’s *d* was used to assess the magnitude of change in EDE scores across all timepoints, with the following interpretation norms: *d* = 0.2 indicated a small effect size, *d* = 0.5 indicated a moderate effect size and *d* = 0.8 indicated a large effect size (Cohen, 1988). Multilevel analysis (MLM) was employed to examine changes over time, as well as predictor variables (Field, 2013; Hox et al., 2017) fit was evaluated using the Akaike Information Criterion (AIC) with models showing a reduction in AIC greater than two considered better (Burnham and Anderson, 2004). A *p*-value of less than 0.05 was considered statistically significant. This threshold was chosen given the theory-driven naturalistic design of the study, as opposed to a randomized controlled trial.

**Figure 1:**
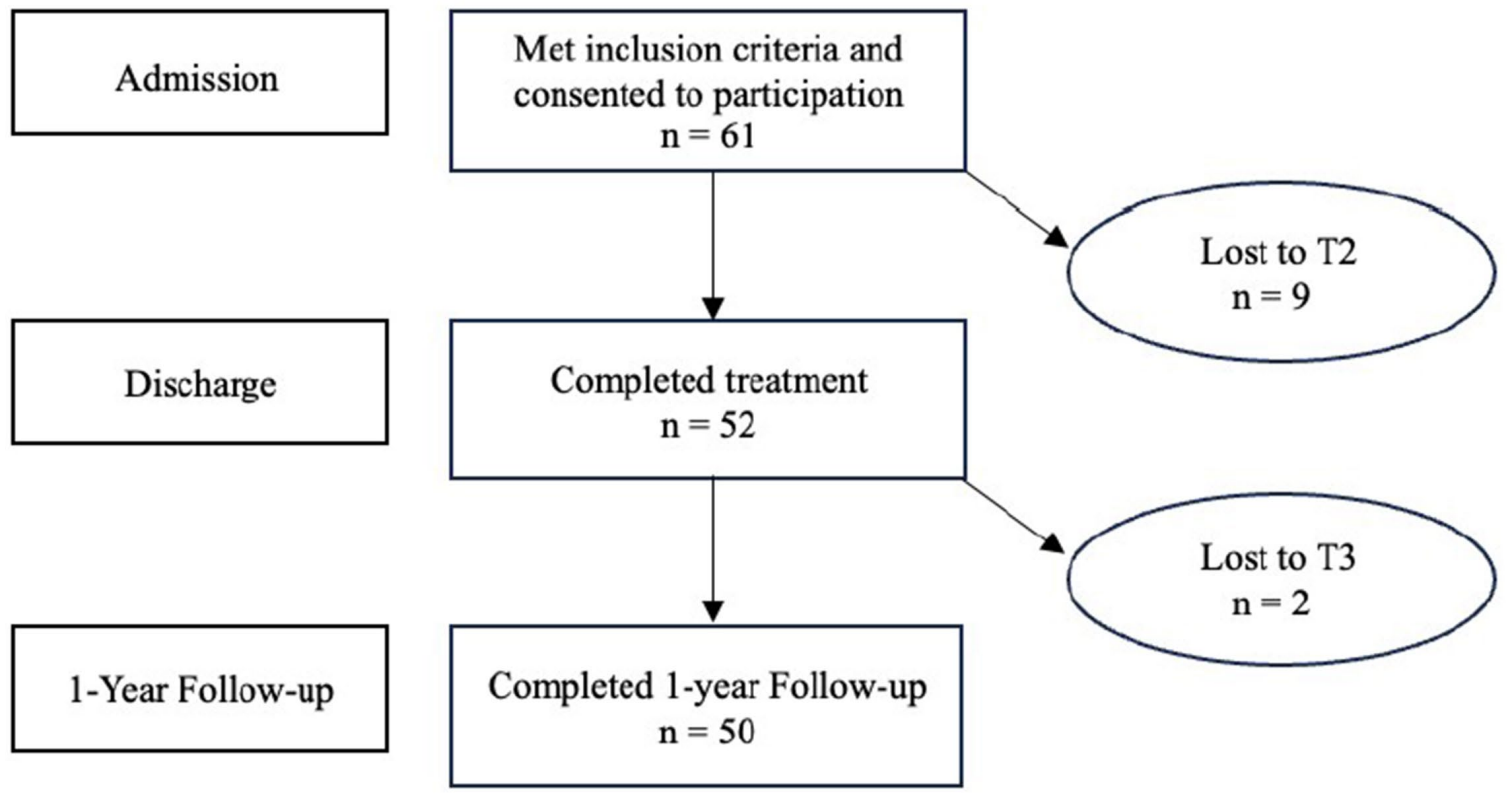
Flow chart illustrating participation rate for all timepoints.

The models were constructed by initially including only a fixed intercept without random effects for each outcome variable (EDE global score as well as subscales “Restriction,” “Eating Concern,” “Weight Concern” and “Eating Concern”). Random intercepts and random slopes were added if they significantly increased model fit, which the random intercept did for all the outcome variables. Test for curvilinearity did not improve model fit, leaving us with a linear growth model for changes over time on all outcome variables. A scaled identity covariance structure of the residuals provided the best model fit for all outcome variables. The first model included only time as a predictor of ED symptoms to examine overall changes. Subsequently, predictors (PTSD and childhood trauma) were added to assess their effect on ED symptoms across the sample. In the second model, we incorporated the two-way interaction effect of time and the predictors to investigate whether covariates influenced changes in ED symptoms over time (Figure 1).

### Ethics

The application was reviewed by the Regional Ethical Committee South-East (REK, reference: 2012/1186). It was determined that the study did not require special approval from REK as the objective was to evaluate an established treatment method, rather than generate new knowledge about health and disease. This study involved no harmful interventions or procedures and was conducted in accordance with current regulations. Furthermore, the study received approval from the data protection officer at Modum Bad, ensuring that all data were anonymized and stored securely in accordance with institutional and national regulations for data privacy and security.

## Results

**Figure 2:**
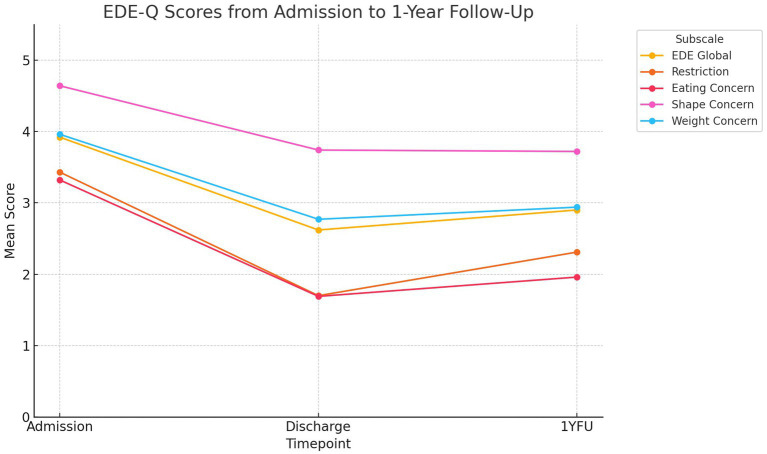
EDE-Q scores from Admisson to 1-year follow-up.

The means and standard deviations for patients in the outcome variables across time are reported in Table 2 and Figure 2. *EDE global* changed at admission 3.92–2.62 at discharge and 2.90 at the 1-year follow-up with effect sizes of *d* = 0.65 from admission to discharge and *d* = 0.20 from discharge to 1-year follow-up. The reduction in EDE subscale scores was largest for restriction, from 3.43 at admission to 2.31 at discharge and 2.31 at 1-year follow-up.

**Table 2:** Mean, standard deviation and effect sizes from pre-to post-treatment and 1-year follow-up (1YFW) on the EDE.

Measures	M (*SD*) Admission	M (*SD*) Discharge	M (*SD*) 1YFU	*d^a^* Admission-Discharge	*d^a^* Discharge-1YFU
EDE global	3.92 (1.11)	2.62 (1.38)	2.90 (1.37)	1.01	0.20
Restriction	3.43 (1.45)	1.70 (1.43)	2.31 (1.71)	1.20	0.38
Eating concern	3.32 (1.50)	1.69 (1.49)	1.96 (1.36)	1.09	0.18
Shape concern	4.64 (1.21)	3.74 (1.49)	3.72 (1.55)	0.65	0.01
Weight concern	3.96 (1.46)	2.77 (1.66)	2.94 (1.56)	0.75	0.11
*N*	61^b^	52^c^	50^d^		

M, mean; SD, standard deviation; EDE, Eating Disorder Examination; 1YFU, 1-year follow-up.

^a^d; Cohen’s *d* = mean1 – mean2/SD collected. ^b^13 missing scores EDE. ^c^8 missing scores EDE. ^d^9 missing scores EDE.

### Hypothesis 1: reduction of ED symptoms over time

The overall level of ED symptoms decreased significantly across time on all outcome measures, EDE global (*β* = −0.43, *p* = <0.001), “Restriction” (*β* = −0.42, *p* = 0.013), “Shape concern” (*β* = −0.37, *p* = 0.005), “Weight Concern” (*β* = −0.43, *p* = 0.002) and “Eating Concern” (*β* = −0.63, *p* = <0.001), confirming our initial hypothesis.

### Hypothesis 2: PTSD and childhood trauma as predictors

Our initial model examined whether the predictors had an effect on the level of ED symptoms over time. No significant main effects of PTSD or childhood trauma on overall symptom levels were found, indicating that these factors were not associated with baseline symptom severity across the outcome measures. However, significant time × group interaction effects were observed. Specifically, PTSD was associated with a slower decrease in “Shape Concern” (*β* = 1.13, *p* = 0.02) and “Weight Concern” (*β* = 1.17, *p* = 0.02) over time, indicating that patients with PTSD exhibited less improvement in these symptoms compared to those without PTSD. Similarly, childhood trauma showed a significant time × group interaction effect on “Shape Concern” (*β* = 0.58, *p* = 0.02), suggesting that patients with childhood trauma also had poorer symptom improvement in this domain across the study period (Tables 3–8).

**Table 3:** Fixed effects estimates (top) and variance (bottom) for models by condition, trauma, and trauma × condition for EDE in multilevel modeling.

Parameters	EDE global	Restriction	Shape concern	Weight concern	Eating concern
Fixed parameters					
Intercept	4.01 (0.30)***	3.29 (0.39)***	4.73 (0.33)***	4.06 (0.35)***	3.58 (0.34)***
Time^a^	−0.43 (0.12)***	−0.42 (0.17)*	−0.37 (0.12)**	−0.43 (0.14)**	−0.63 (0.14)***
Random parameters					
Residual	0.94 (0.17)***	1,90 (0.35)***	1.01 (0.19)***	1.16 (0.21)***	1.28 (0.23)***
Intercept	0.86 (0.27)***	0.71 (0.37)	1.22 (0.36)***	1.45 (0.42)***	0.86 (0.31)**
AIC	352.00	402.22	368.36	383.66	375.35

EDE, Eating Disorder Examination; AIC, Akaike Information Criterion.

a Time = 0, 1, 2.

*p < 0.05. **p < 0.01. ***p < 0.001.

**Table 4:** Childhood trauma and PTSD as predictors for change in EDE global over time.

Parameters	EDE global	EDE global	EDE global	EDE global
	Model 1^a^	Model 2^b^	Model 1^a^	Model 2^b^
			Fixed parameters	
Intercept	3.85 (0.33)***	4.08 (0.39)***	4.05 (0.31)***	4.15 (0.32)***
Time^c^	−0.42 (0.12)***	−0.53 (0.15)***	−0.44 (0.12)***	−0.49 (0.12)***
CT	0.40 (0.35)	−0.18 (0.62)		
Time*CT		0.29 (0.25)		
PTSD			−0.31 (0.63)	−1.45 (0.99)
Time*PTSD				0.71 (0.46)
			Random parameters	
Residual	0.93 (0.17)***	0.92 (0.17)***	0.95 (0.17)***	0.90 (0.17)***
Intercept	0.86 (0.27)**	0.88 (0.28)**	0.87 (0.28)**	0.95 (0.30)**
AIC	350.93	350.55	350.86	348.28

Parenthesise = standard error. CT, Childhood trauma; PTSD, Post-traumatic stress disorder; AIC, Akaike Information Criterion.

^a^Model 1 = Main effect of time and predictor variable, ^b^Model 2 = Predictor × time, ^c^Time = 0, 1, 2.

*p < 0.05. **p < 0.01. ***p < 0.001.

**Table 5:** Childhood trauma and PTSD as predictors for change in Restriction over time.

Parameters	Restriction	Restriction	Restriction	Restriction
	Model 1^a^	Model 2^b^	Model 1^a^	Model 2^b^
			Fixed parameters	
Intercept	3.12 (0.43)***	2.99 (0.50)***	3.29 (0.41)***	3.32 (0.42)***
Time^c^	−0.41 (0.17)*	−0.35 (0.21)	−0.42 (0.17)*	−0.44 (0.17)*
CT	0.40 (0.39)	0.76 (0.80)		
Time*CT		−0.17 (0.35)		
PTSD			−0.07 (0.70)	−0.38 (1.30)
Time*PTSD				0.19 (0.55)
			Random parameters	
Residual	1.90 (0.35)***	1.94 (0.36)***	1.90 (0.36)***	1.92 (0.37)***
Intercept	0.70 (0.38)	0.67 (0.38)	0.74 (0.39)	0.75 (0.40)
AIC	401.21	401.24	401.09	400.04

Parenthesise = standard error. CT, Childhood trauma; PTSD, Post-traumatic stress disorder; AIC, Akaike Information Criterion.

^a^Model 1 = Main effect of time and predictor variable, ^b^Model 2 = Predictor × time, ^c^Time = 0, 1, 2.

*p < 0.05. **p < 0.01. ***p < 0.001.

**Table 6:** Childhood trauma and PTSD as predictors for change in Eating concerns over time.

Parameters	Eating concerns	Eating concerns	Eating concerns	Eating concerns
	Model 1^a^	Model 2^b^	Model 1^a^	Model 2^b^
			Fixed parameters	
Intercept	3.48 (0.37)***	3.58 (0.44)***	3.59 (0.35)***	3.63 (0.36)***
Time^c^	−0.62 (0.14)***	0.67 (0.18)***	−0.63 (0.14)***	−0.65 (0.15)***
CT	0.23 (0.37)	−0.03 (0.70)		
Time*CT		0.13 (0.30)		
PTSD			−0.13 (0.67)	−0.52 (1.12)
Time*PTSD				0.24 (0.54)
			Random parameters	
Residual	1.28 (0.23)***	1.30 (0.24)***	1.29 (0.23)***	1.30 (0.24)***
Intercept	0.89 (0.31)**	0.88 (0.31)**	0.89 (0.32)**	0.89 (0.32)**
AIC	375.10	375.55	374.28	373.48

Parenthesise = standard error. CT, Childhood trauma; PTSD, Post-traumatic stress disorder; AIC, Akaike Information Criterion.

^a^Model 1 = Main effect of time and predictor variable, ^b^Model 2 = Predictor × time, ^c^Time = 0, 1, 2.

*p < 0.05. **p < 0.01. ***p < 0.001.

**Table 7:** Childhood trauma and PTSD as predictors for change in Shape concerns over time.

Parameters	Shape concerns	Shape concerns	Shape concerns	Shape concerns
	Model 1^a^	Model 2^b^	Model 1^a^	Model 2^b^
			Fixed parameters	
Intercept	4.54 (0.36)***	5.00 (0.41)***	4.76 (0.34)***	4.91 (0.34)***
Time^c^	−0.36 (0.13)**	−0.57 (0.15)***	−0.37 (0.13)**	−0.44 (0.13)***
CT	0.47 (0.40)	−0.72 (0.05)		
Time*CT		0.58 (0.25)*		
PTSD			−0.31 (0.71)	−2.10 (1.05)*
Time*PTSD				1.13 (0.47)*
			Random parameters	
Residual	1.01 (0.18)***	0.92 (0.17)***	1.02 (0.19)***	0.90 (0.17)***
Intercept	1.22 (0.36)***	1.30 (0.37)***	1.23 (0.37)***	1.38 (0.40)***
AIC	366.99	362.75	367.01	361.34

Parenthesise = standard error. CT, Childhood trauma; PTSD, Post-traumatic stress disorder; AIC, Akaike Information Criterion.

^a^Model 1 = Main effect of time and predictor variable, ^b^Model 2 = Predictor × time, ^c^Time = 0, 1, 2.

*p < 0.05. **p < 0.01. ***p < 0.001.

**Table 8:** Childhood trauma and PTSD as predictors for change in Weight concerns over time.

Parameters	Weight concerns	Weight concerns	Weight concerns	Weight concerns
	Model 1^a^	Model 2^b^	Model 1^a^	Model 2^b^
			Fixed parameters	
Intercept	3.89 (0.39)***	4.18 (0.45)***	4.16 (0.36)***	4.32 (0.36)***
Time^c^	−0.42 (0,14)**	−0.56 (0.17)**	−0.45 (0.14)**	−0.52 (0.13)***
CT	0.44 (0.43)	−0.29 (0.72)		
Time*CT		0.36 (0.28)		
PTSD			−0.83 (0.76)	−2.68 (1.13)*
Time*PTSD				1.17 (0.50)*
			Random parameters	
Residual	1.15 (0.21)***	1.13 (0.21)***	1.17 (0.22)***	1.04 (0.20)***
Intercept	1.46 (0.42)***	1.48 (0.43)***	1.41 (0.42)***	1.60 (0.46)***
AIC	382.45	381.51	381.19	375.81

Parenthesise = standard error. CT, Childhood trauma; PTSD, Post-traumatic stress disorder; AIC, Akaike Information Criterion.

^a^Model 1 = Main effect of time and predictor variable, ^b^Model 2 = Predictor × time, ^c^Time = 0, 1, 2.

*p < 0.05. **p < 0.01. ***p < 0.001.

## Discussion

This study examined the trajectories of ED in mothers from the start of treatment through to the 1-year follow-up. Consistent with our hypotheses, the overall level of ED symptoms significantly decreased over time. In the second part of our analyses, we found that both PTSD and childhood trauma were significant predictors of poorer outcomes for symptoms for “Shape Concern,” while PTSD also predicted worse outcomes for “Weight Concern.”

### Hypothesis 1: reduction of ED symptoms over time

Analysis of the effects demonstrate that the greatest change in symptoms occurred during treatment (*d* = 1.01), followed by a small, non-significant increase in symptoms between discharge and the 1-year follow-up (*d* = 0.20). This indicates that symptoms remained relatively stable post-treatment, with no significant worsening observed at follow-up. Given that this study focuses on symptom trajectories we cannot attribute this change solely to the treatment, and a randomized controlled trial would be necessary to establish causal effect. Nevertheless, the symptom changes observed during this study align with findings form recent literature review and meta-analysis which conclude that the CBT framework is an effective approach for reducing ED symptoms (Atwood and Friedman, 2020; Dahlenburg et al., 2019; de Jong et al., 2018). Similar reductions in symptoms have been demonstrated in transdiagnostic samples (Byrne et al., 2011; Signorini et al., 2018) and within naturalistic settings (Byrne et al., 2011; Knott et al., 2015). The analyses further indicated that the changes were sustained over time. This contributes to the growing body of longitudinal research that have reported lasting reduction in ED symptoms following treatment for AN (Calugi et al., 2017; Fichter et al., 2017), BN (Poulsen et al., 2014; Wonderlich et al., 2014) and in transdiagnostic samples (Fairburn et al., 2015; Signorini et al., 2018). While this study demonstrates sustained improvement at 1-year follow-up, the long-term course of recovery beyond this point remains unknown. Future research should aim to include extended follow-up periods (e.g., 5–10 years) to assess the durability of symptom change and identify factors that promote or hinder long-term recovery in mothers with ED.

Conducting analyses at the subscale level can provide valuable insights into the trajectories of psychological and behavioral symptoms of ED, offering a more nuanced understanding of remission (Clausen, 2004). At the subscale level we observed significant reductions across all EDE subscales: “Restriction,” “Shape Concern,” “Weight Concern,” and “Eating Concern” from admission to 1-year follow-up. A recent meta-analysis of RCT studies examining the effect of CBT on the “Shape Concern” and “Weight Concern” concluded that CBT is effective in reducing these types of cognitions, though our understanding of symptom trajectories in the context of treatment remains limited (Linardon, 2018). In this study “Shape Concern” exhibited the smallest reduction and the lowest effect size, followed by “Weight Concern.” These findings are consistent with those of Clausen (2004) who noted that behavioral symptoms of AN and BN remitted earlier than cognitions related to shape and size. This is further supported by research emphasizing the critical role of disturbed body-related cognitions in the treatment of AN (Glashouwer et al., 2019) and BN (Helverskov et al., 2010), as well as evidence indicating that body image and body disturbances often persist after treatment (Engel and Keizer, 2017; Eshkevari et al., 2014; Exterkate et al., 2009).

Several characteristics for this sample, for example extensive prior treatment and high levels of comorbidities, are associated with a poorer prognosis (Steinhausen, 2002; Steinhausen and Weber, 2009; Vall and Wade, 2015). Our study indicates that also inpatient populations experience symptom reduction during the treatment period, and this reduction in symptoms is maintained, something that is in accordance with earlier studies (Danielsen et al., 2020; Rø et al., 2005; Vrabel et al., 2010). This is important, particularly given the longevity of illness and the participants meeting criteria for inpatient level of care. The observed change gives hope for a patient population that by many is considered difficult to treat (Steinhausen, 2002; Treasure et al., 2020). At the same time the mean symptom levels at both discharge and at 1-year follow-up are still above the clinical cut-off for ED (Rø et al., 2015). Residual symptoms after treatment are not uncommon (Tomba et al., 2019), but problematic since it can increase the risk for worsening of symptoms and relapse (Vall and Wade, 2015). Further clinical investigations of treatment and remission are therefore warranted.

### Hypothesis 2: PTSD and childhood trauma as predictors

In our study, trauma experiences of showed predictive value at the EDE subscale level. Both PTSD and childhood trauma predicted worse outcomes for “Shape Concern,” and PTSD also predicted worse outcomes for “Weight Concern.” To our knowledge, no previous studies have examined PTSD and/or childhood trauma as predictors for ED symptoms at a subscale level. However, our findings align with prior research demonstrating that trauma predicts poorer treatment trajectory in ED (Hazzard et al., 2021; Mitchell et al., 2016; Trottier, 2020, s. 2; Vrabel et al., 2010). In our study, PTSD was a significant predictor of worse outcomes in both Shape Concern and Weight Concern. This aligns with Hazzard et al. (2021), who found that patients with both childhood trauma and comorbid PTSD had worse outcomes than those with childhood trauma alone. A possible explanation is that PTSD signifies more severe symptoms of trauma, which is linked to more serious ED pathology (Tagay et al., 2014).

Consistent with earlier findings (Clausen, 2004), “Shape Concern” and “Weight Concern” showed the least change in our study. Further elucidating our findings, it is important to consider the specific challenges faced by mothers in relation to body image. Pregnancy, childbirth, and breastfeeding are accompanied by significant physical changes, and many women experience heightened body dissatisfaction during the postpartum period. Research has shown that mothers are particularly vulnerable to shape- and weight-related concerns in the months following childbirth, which can in turn exacerbate or maintain disordered eating behaviors (Clark et al., 2009; Meireles et al., 2015). In addition, societal expectations around rapid postpartum recovery and unrealistic standards for women’s bodies can place additional pressure on mothers, particularly those with a history of ED. Recent research suggests that the combination of physical transformation and sociocultural pressures may contribute to the persistence of body-related cognitions, such as concerns about weight and shape, during and after treatment. For instance, a systematic review by Song et al. (2023) demonstrated that exposure to Western cultural ideals and acculturation-related stress are strongly associated with enduring body image concerns. These factors may help explain why shape and weight concerns showed smaller improvements in our study compared to other symptom domains. Predictive analyses suggest that exposure to trauma may also explain these limited changes. This is of importance because an obsession with shape and weight is theorized as possible maintaining mechanisms for dysfunctional eating behavior (Fairburn et al., 2003). For example, it is documented that the degree to which one can reduce concern about shape and weight during treatment is directly related to degree of reduction in overeating and compensatory behavior (Linardon, 2018). It has also been shown that less worry about shape and weight predicts better outcomes of treatment in patients with ED (Vall and Wade, 2015). Traumatic experiences might in other words predict increased likelihood of a worse treatment outcome through a specific association to shape and weight related cognition. The finding that trauma did not have a predictive effect on overall ED pathology (EDE global), but still significantly predicted trajectories in ED-related cognitions (subscale) underscores the importance of individually assessing the distinct symptoms that constitute an ED. In a population marked by complexity and high degrees of comorbidity, assessing specific cognitive and behavioral symptoms may provide more informative insights than relying solely on global symptom measures.

### Motherhood and ED—clinical implications

Although this study did not directly investigate parenting skills or family dynamics, these aspects are integral to the treatment context and warrant consideration in interpreting the clinical implications for mothers with ED. It remains unclear whether mothers with ED differs from the broader patient population in terms of ED symptom development. It is possible that having children is associated with a reduction in eating problems (Fichter et al., 2017; von Soest and Wichstrøm, 2008), but the long-time effects are uncertain (Tabler and Utz, 2020). Few studies have explored how motherhood might influence ED symptomatology (Tabler and Utz, 2020; von Soest and Wichstrøm, 2006, 2008). What is known is that parenthood is seldom addressed in ED treatment (Kaspersen and Rø, 2012), although mothers with ED report high concern for their children, and many worry whether they are good enough parents (Stitt and Reupert, 2014; Tuval-Mashiach et al., 2013). This might however not be addressed during treatment due to shame and fear for the potential legal consequences of disclosing perceived inadequacy as a parent. Therapists might also be reluctant to ask, in fear of disrupting the working alliance (Fleming et al., 2022). Subsequently, important themes related to parental responsibilities, struggles and -identity, specific to mothers with serious ED, might remain unexplored in treatment without this explicit focus. It remains however an important clinical challenge to disentangle exactly what effective, relevant and feasible treatment looks like for this population, who reports a generally high level of symptom burden and where trauma experiences are not uncommon.

One approach that warrants further clinical attention seems to be the possibility of sequential treatment. Another notable strength is the brief, flexible format. For example, the NURTURE pilot (Runfola et al., 2014) achieved 100% retention using only 16 weekly web-based group sessions, making it feasible for mothers balancing caregiving and treatment. Alongside evidence from studies like Stein et al. (2006)—which found that short-term inpatient video-feedback interventions enhanced mother–infant interactions—this supports the case for high-intensity, brief interventions that promote engagement and reduce dropout among mothers with young children. Our analyses indicate that patients can gain an equally large symptom reduction from sequential treatment as compared to continuous inpatient stays. Especially for mothers with ED a sequential treatment program might be practical, since the parental responsibilities make long absences from home problematic (Kaspersen and Rø, 2012). In our sample, the mean age of the children was reported to be 8.5 years, suggesting that the majority are likely to live at home and rely on consistent parental care and supervision (Bryant-Waugh et al., 2007). Sequential treatment approaches might ease the practical and emotional burden of long absences. This may encourage patients to complete treatment and enhance therapeutic engagement during in-patient weeks. The involvement of the family, goal-oriented feedback on the mother–child interactions and a relational framework in therapy are also likely to be key components in specialized intervention for this group. Additionally, increased awareness of how ED may impact children could also serve as a motivating factor for treatment adherence (Patel et al., 2002).

An important point with possibly far-reaching consequences is raising awareness of how parents act as models for their children when it comes to attitudes and habits concerning food, eating and body image (Davison and Birch, 2001; Lowes and Tiggemann, 2003). It is crucial that this part of the therapeutic work is done by professionals with experience and competence in how to facilitate insight into these transmissional effects, without inflicting unnecessary shame and guilt (Winnicott, 1960; Zerbe, 1993). A note to this is that our study showed that the subscales of “Restriction” and “Easting Concerns” showed the greatest reduction, for which there can be several possible explanations. One possibility is that the patients commit to a meal plan during their stay (Modum Bad, 2013) and that this reduced the anxious thoughts concerning food, as well as prevent restrictive behavior during the stay. Another possible explanation is the explicit focus on normalizing eating behavior during treatment. Helping patients normalize their eating behavior might mitigate the risk of overt ED behavior that affects the family dynamics. That is, it might help mothers to contain their own challenges, thereby strengthening parental functioning and flexibility as well as modeling more healthy eating patterns for their children. This in turn, might decrease risk of generational transmission and improve the general family dynamic (Gerhardt, 2014).

Evidence to date has shown that ED is associated with disturbance in general parental functioning, as well as ED specific problems in parent–child interactions, but we need more research covering other developmental stages than the infant/toddler period. It is likely that the difficulties faced by mothers with ED will change and vary over time as their children grow older, and being a parent to older children or teenagers might pose age-specific challenges. On this note, it is an important point that increased awareness for how their disorder affect their children can be an important motivational factor on the path toward recovery (Taborelli et al., 2016). Mothers with ED are doing their utmost to provide and care for their children, and incorporating an understanding of the parent role into treatment compliments this line of thought. Strengthening parental skills for those who struggle in the role, as well as increasing awareness for generational transmission of strained perceptions of food, eating and body image, are potentially important and impactful elements in therapy. Focusing treatment on the parental aspects of ED related challenges might have an important preventative potential, something that should be the topic of future clinical investigations (Orbach and Rubin, 2014).

### Strengths, limitations and future directions

The dataset is from the period of 2011–2013 and is therefore slightly dated. However, there exists no other study currently of the effectiveness of treatment for mothers with ED to our knowledge, both bringing value to our findings and accentuating the need for further studies. Furthermore, the treatment protocols at the hospital from which this dataset originates have remained consistent, enhancing our findings relevance and representativeness for current practices. Another limitation is the relatively small sample size (*N* = 61), something that can weaken the strength of the statistical tests. Moreover, the subgroup sizes for PTSD and childhood trauma were relatively small, which may have limited the statistical power to detect some effects and interactions related to trauma. The possibility cannot be excluded that more connections could be discovered in a larger sample. At the same time the low N indicate that this is trustworthy significant findings that with all probability can be replicated.

It is possible that splitting the trauma patients into two groups (PTSD and childhood trauma) creates an artificial divide which hampers the statistical power to demonstrate the real predictive effect of trauma in general for the trajectory of ED. Nonetheless, it still seems of crucial importance to examine whether patients have trauma experience, since we know that this is common among individuals with ED (Hudson et al., 2007; Molendijk et al., 2017), is related to the severity of ED (Kong and Bernstein, 2009; Mitchell et al., 2016) and to the tailoring of ED treatment. Although this study focused specifically on the predictive value of PTSD and childhood trauma, we acknowledge that other factors—such as socio-economic status, social support, and access to external resources—may also influence treatment outcomes. Future studies should consider including such contextual variables to provide a more comprehensive understanding of the mechanisms shaping recovery.

Among the strengths of this study is the data collection, which was done at a highly specialized unit for ED at Modum Bad, an institution with long experience in treatment and psychotherapy research. The level of ED symptoms was measured at three different timepoints, and the measurement tools are considered to have good psychometric qualities. The time perspective in the study allows us to say something about the long-time effect of the treatment in a reliable way. As a starting point for analyses, we have used the Intention-to-treat-approach (Gupta, 2011), meaning that all participants were included in the analyses, independently of how much treatment they completed. This principle reduces the risk of bias in the study, for example related to drop out bias, and gives us a more valid presentation of change over time (McCoy, 2017).

An important question is whether the results can be generalized to a greater population. The study was conducted in a Norwegian context within a publicly funded specialist inpatient unit. While this increases ecological validity within similar welfare-based healthcare systems, it may limit direct transferability to countries with different healthcare structures or access constraints. The treatment itself was delivered in a high-intensity, sequential inpatient format, which is not widely available in all settings. However, this flexible structure may serve as a valuable model for adaptation in services seeking to increase accessibility for mothers. The participants also represent a selected group with a high degree of symptom severity. High levels of comorbidity, long mean time duration of illness and a high number of treatment attempts is not representative for all people with ED. It has been observed however, that serious symptoms and high degree of complexity is not uncommon in ordinary outpatient clinical settings (Haas and Clopton, 2003; Palmer, 2006). Additionally, the analyses revealed a significant in between person variance at admission, something that indicates high degree of heterogeneity in the sample. This is representative for the ED population, strengthening the transfer value.

## Conclusion

There are still large gaps in the evidence base regarding mothers with ED (Reinar et al., 2015). Unanswered questions relate to both understanding how aspects of motherhood and ED influence each other, as well as to how we best address specific challenges that arise in the treatment of mothers with ED. Our analysis indicate that mothers might benefit on treatment programs that incorporate an understanding of their parental role and challenges that arise in the context of struggling with ED while having responsibility for children. While our study did not directly measure parental skills or caregiving outcomes, these clinical implications are drawn from the treatment context and existing literature, suggesting relevant areas for future research and clinical focus. Sequential frameworks for in-patient stays might be a feasible approach, but further studies are needed to disentangle efficiency regarding symptom reduction. As this was a naturalistic study without a control group, causality cannot be inferred. The observed symptom reductions may be influenced by other unmeasured factors such as spontaneous remission, external support, or concurrent treatments. While the longitudinal design offers valuable insight into symptom trajectories, future research should incorporate both RCTs and qualitative methods to determine efficacy and illuminate mechanisms of change—particularly in relation to parental roles and trauma histories. As our results show, conducting analysis at a subscale level might reveal nuances in symptom trajectories that are lost when looking at global scores. Finally, our study confirms existing evidence that emphasize the role of trauma in ED, expanding upon its predictive value when examining symptom change over time. There is a great need for more prospective and longitudinal studies of specialized treatment and the symptom trajectories of mothers with ED, to shed light on the heterogeneous and non-linear process of change and recovery.

## Data Availability

The raw data supporting the conclusions of this article will be made available by the authors without undue reservation.
